# The insulin market reaches 100

**DOI:** 10.1007/s00125-022-05680-y

**Published:** 2022-03-11

**Authors:** David Beran, Edwin A. M. Gale, John S. Yudkin

**Affiliations:** 1 Division of Tropical and Humanitarian Medicine, Faculty of Medicine, University of Geneva and Geneva University Hospitals, Geneva, Switzerland; 2 University of Bristol, Bristol, UK; 3 Division of Medicine, University College London, London, UK

**Keywords:** Diabetes, Health services accessibility, Insulin

## Introduction

The coronavirus disease-2019 (COVID-19) pandemic showed that private companies can move from discovery to large scale production with amazing speed. ‘The reason we have the vaccine success is because of capitalism, because of greed’, claimed British Prime Minister Boris Johnson [1], rightly pointing out that industrial investment is needed to bring scientific advance to the bedside, but forgetting the decades of public investment in research which made this possible. The story of insulin has often been told, but the way in which an unprepared academic institution handled the far-reaching commercial implications of its discovery is rarely mentioned, and may be relevant to a world in which up to 60% of users lack secure access to affordable insulin [2].

George Clowes of Eli Lilly advised the University of Toronto to take out a defensive patent on insulin in order to forestall interlopers intent on profit, and thyroxine provided the model for Toronto’s Insulin Committee. Edward Kendall’s 1914 patent for this was shared with the Mayo brothers and transferred to the University of Minnesota on the condition that it supervised commercial exploitation of the discovery. Since the chemical nature of insulin was unknown, the patent could only be defined in terms of its method of extraction and properties, and Kendall advised Macleod to patent both the method and its clinical application, which risked that both elements might be superseded by subsequent patents [3]. The Canadian patent was assigned to Charles Best and James Collip and transferred to the University of Toronto for the princely sum of one (Canadian) dollar each. John Macleod and Frederick Banting believed that it would be contrary to the Hippocratic Oath for them to be named, and their opinion was summed up in Banting’s remark that ‘insulin belongs to the world, not to me’ [4].

The Insulin Committee believed that high quality insulin should be made available worldwide and at an affordable price; manufacturers who could meet quality standards and were prepared to pool knowledge were freely licensed to produce it. The benefits of commercial collaboration soon became clear, for Collip’s extraction method was extremely inefficient, with a yield of 15–40 units per kg of beef pancreas. George Walden of Eli Lilly increased the yield 10- to 100-fold by introducing isoelectric focusing in the autumn of 1922. This was the discovery that made insulin commercially viable and Clowes, carried away by enthusiasm, announced that ‘we can produce in Indianapolis a sufficient quantity of Iletin (Iletin was the first trade name of insulin sold by Eli Lilly) to supply the entire needs of the civilised world’ [3]. Free intellectual property made insulin a ‘gentleman’s market’, and companies vied to make it available. The first vials marketed for around Canadian (CAD)$1, equivalent to ~CAD$12 in 2006, but the price soon came down [5] with insulin produced by Eli Lilly sold for 3.5% of its initial price in the 1930s [6].

## The high price of innovation

The days of animal insulin were numbered when biosynthetic human insulin reached the market in the 1980s. This stunning intellectual achievement resulted from research undertaken in publicly funded academic institutions, but the shape of things to come was seen when Genentech won the race to synthesise a human gene. History was made when a company founded by Boyer and Swanson with an initial investment of US$500 each raised US$66 million on the day of its launch in October 1980 [7]. Francis Crick famously said that ‘DNA makes RNA makes protein’, but academics now learned that genes make proteins make money, and research into biopharmaceuticals would never be the same.

The new technology priced smaller manufacturers out of the market, and many were taken over or closed down. The resulting ‘Big Three’—Eli Lilly, Novo Nordisk and Sanofi—had acquired >96% of the global market by the start of the twenty-first century [8]. Market control coincided with a commercial incentive to move on from the human molecule, which cannot be patented, to genetically engineered analogues, which can. Commercial logic was matched by clinical enthusiasm, for restructuring the insulin molecule appeared to promise unlimited possibility. However, evolution has optimised insulin’s binding site with its receptor to the point that it is closely identical in many species. This left genetic engineers with limited scope for improvement, and their options were therefore limited to modifications which affect insulin’s absorption and bioavailability.

## The ‘new insulins’ are modified delivery systems

Subcutaneous insulin has inescapable limitations, for absorption is slow and erratic and insulin is fed into the systemic, rather than the portal, circulation. These limitations cannot be overcome by engineering the molecule to speed up or slow down its absorption, and the analogue insulins are essentially modified delivery systems. The role of analogue insulin in diabetes management remains contentious. The 2021 ADA/EASD guidelines make no distinction between human and analogue insulin [9]. The WHO added long-acting analogues to its Essential Medicines List in its 2021 revision, counter to evidence it had previously found convincing [10, 11]. Some people undoubtedly benefit from their use, but across-the-board advantage in terms of glycaemic control has yet to be demonstrated [12]. The main objection to the analogues (‘modern’ insulins in sales jargon) is cost—typically around six times the cost of human insulin [13]. Cost apart, there is no very good reason not to use them.

The most striking feature of the insulin market is the speed with which it expanded from a value of around US$2 billion in 1995 to US$3 billion in 2000, equivalent to a compound annual growth rate (CAGR) of 8%. It then jumped to an estimated US$7.3 billion in 2005, equivalent to a CAGR of 19% [14], and the ‘Big Three’ were grossing US$19.9 billion (uncorrected US$ figures) in insulin sales by 2020. Around half of these sales were by Novo Nordisk, with Eli Lilly and Sanofi around one quarter each [15–17]. The three companies differ in their dependence on insulin for total sales (Table 1). Many biosimilar manufacturers have the potential to enter the market today, but the regulatory barriers to entry have generally proved too high, and the ‘Big Three’ have produced biosimilars of their own at almost the same price as the branded product [18].

**Table 1:** Total turnover, turnover linked to diabetes and turnover linked to insulin for 2020 for Eli Lilly, Novo Nordisk and Sanofi

Company	Turnover (US$ billion)	Percentage of turnover linked to diabetes	Percentage of turnover linked to insulin
Eli Lilly	24.5	46.1%	20.4%
Novo Nordisk	20.6	85%	44.5%
Sanofi	43.5	~14.5%	13.1%

## Why did the market expand so rapidly?

A major reason for this rapid market expansion was the speed with which insulin came to be used in type 2 diabetes, once known as non-insulin-dependent diabetes. In the UK, for example, 2.4 people per 1000 of the adult population used insulin in 1991, and 0.7/1000 of these were considered to have type 2 diabetes. By 2010 the number of insulin users had increased to 6.7/1000, and 4.3/1000 of them were deemed to have type 2 diabetes; a sixfold increase [19].

The Diabetes Control and Complications Trial (DCCT) confirmed the value of glycaemic control in type 1 diabetes, and the UK Prospective Diabetes Study (UKPDS) is widely assumed to have done the same for type 2 [20]. This it did, but with important qualifications. Improved glucose control did not improve survival or quality of life, nor did it affect shorter term cardiovascular mortality. Early introduction of insulin in type 2 diabetes has yet to show conclusive benefit in comparison with other glucose-lowering therapies, whereas both the blood pressure control arm of UKPDS [20] and the Steno-2 study [21] showed unequivocal evidence of ‘patient outcomes that matter’ within the trial period. Such clear evidence notwithstanding, industry-supported educational activities, major conferences and research publications all display an unremitting focus upon glucose control.

## The challenge of insulin’s affordability

Biosynthesis ended fears of a shortage of animal pancreas, and has made insulin cheap to produce: human insulin has an estimated production cost of US$2.28–3.37 per 1000 unit vial [22]. Allowing for 30% profit, a year’s supply for someone who takes 40 units per day should cost less than US$71 per year. The equivalent maximum for analogues other than detemir would be US$133. How come insulin remains out of reach for many worldwide?

Regarding affordability, the price of a vial of Humalog (lispro) insulin in the USA increased from US$21 in 1999 to US$322 in 2019 [23], a rise of 1500% that was closely ‘shadowed’ by the other manufacturers. The producers are frequently blamed, but much of the profit is made by middlemen along the supply chain leading up to the pharmacy counter [24]. Doug Langa, North American chief executive officer (CEO) of Novo Nordisk, pointed out in testimony to Congress in 2019 that a vial of his company’s human insulin could be obtained in Walmart for US$25, or US$1 per day for the average user [25]. Americans can afford human insulin; it is analogue insulin that is unaffordable.

Elsewhere in the world, people in low- and middle-income countries (LMIC) who buy their own insulin are charged at rates commensurate with the costs of production, US$35.40 on average for a year’s supply via the public sector, for example [13]. Others may even be able to get insulin for free provided by the health system. The problem is that insulin is often unavailable in the public sector and must be purchased in the private sector at a much higher price. Paradoxically, people in the world’s richest country struggle to pay for their insulin, whereas those in poorer countries may not be able to obtain it at all.

## The role of the market

In theory, the market should provide the most cost-effective solution for problems of distribution, but the market for insulin is self-evidently not open, and competition is most intense at the ‘high end’ of the market, where Americans pay five to ten times as much for their insulin as anyone else [26]. This means that there is little commercial incentive to provide insulin to users in poorer parts of the world.

What then awaits us in the second century of the insulin market? There is little point in only blaming the manufacturers, for the inflated price of insulin also reflects the greed of intermediaries along the supply chain as well as that of shareholders. Added costs of diabetes include consumables such as syringes, needles and pens, and the manufacturers of test strips are among the most shameless profiteers [27]. Affordable insulin does, however, need commercial partners, and the challenge for the future will be to make this partnership work better. This is far from the intentions of the people who gave us insulin and is unacceptable to those who consider access to insulin a basic human right.

## How might the ‘Big Three’ view the future?

The traditional approach to market scrutiny is a SWOT (strengths, weaknesses, opportunities and threats) analysis. Strengths include an ever-growing number of people who need insulin, uncontested market dominance and a complaisant medical profession. The key weakness is that the fundamental limitations of subcutaneous insulin cannot be overcome by genetic engineering, and marginal improvements in absorption face diminishing returns. Opportunities for the future include ‘smart’ insulins linked to feedback control of blood glucose or forms of insulin which act selectively on the liver, but these ‘high end’ prospects, even if realised, are unlikely to benefit the average user.

Meanwhile, threats accumulate. The license to manufacture popular analogues such as glargine has expired, and biosimilars are slowly becoming available. Large manufacturers are currently protected by the high cost of regulatory approval (the WHO prequalification scheme for insulin is designed to address this) and the even higher cost of entering the market. It is, however, hard to defend a market position in which your product is far more expensive than that of your competitors, and in the absence of striking new developments, this is likely to become increasingly evident with the passage of time. The trend, therefore, is to diversify towards other segments of the market. Of the ‘Big Three’, only Novo Nordisk has a majority financial focus on diabetes, and the company’s interest in incretins has moved towards their application to obesity [15].

On current trends, the boom in insulin treatment of type 2 diabetes is ending, and other therapies are becoming more profitable. There will always be a need for insulin especially for people with type 1 diabetes, but genetic engineering of subcutaneously administered insulin might (or might not) be approaching its limits. Advances in technology and transplantation are likely to determine the future of type 1 diabetes in affluent countries, but equity is a major consideration. In a world where, as of February 2021, ten countries had appropriated 75% of all COVID-19 vaccines, and 130 nations had no vaccine at all [28], quality assured, affordable insulin is what countries need, together with the health infrastructure to deliver it. Insulin and its analogues are relatively inexpensive, and the supply is potentially inexhaustible. Market forces have inflated its cost, and market forces, if allowed free play, could well drive it down again. This, ultimately, is a matter for the politicians. Should it ever be allowed to happen, a free market in insulin could turn out to be part of the solution rather than part of the problem.

## Abbreviations

CAGR: Compound annual growth rate
UKPDS: UK Prospective Diabetes Study
